# Improved Esterification of Citric Acid and n-Butanol Using a Dense and Acid-Resistant Beta Zeolite Membrane

**DOI:** 10.3390/membranes12121269

**Published:** 2022-12-15

**Authors:** Zhengquan Yang, Mingyu Peng, Yu Li, Xiaowei Wu, Tian Gui, Yuqin Li, Fei Zhang, Xiangshu Chen, Hidetoshi Kita

**Affiliations:** 1 State-Province Joint Engineering Laboratory of Zeolite Membrane Materials, Institute of Advanced Materials (IAM), Jiangxi Normal University, Nanchang 330022, China; 2 Environmental Science and Engineering, Graduate School of Sciences and Technology for Innovation, Yamaguchi University, Ube 755-8611, Japan

**Keywords:** improved esterification, Tributyl citrate, beta zeolite membrane, PV-esterification, high conversion

## Abstract

In this work, a dense and acid-resistant beta zeolite membrane was applied to improve the esterification of citric acid and n-butanol, for the first time. Through the continuous removal of the by-product water via pervaporation (PV), the conversion of citric acid was significantly enhanced from 71.7% to 99.2% using p-Toluenesulfonic acid (PTSA) as catalyst. PTSA was a well-known strong acid, and the membrane kept almost no change after PV-esterification, indicating the superior acid resistance of beta zeolite membrane. Compared to the use of acid-resistant MOR zeolite membrane by PV-esterification, a consistently higher conversion of citric acid was obtained using a high-flux beta zeolite membrane. The results showed that high water permeation on the beta zeolite membrane, with good acid resistance, had a strong promoting effect on esterification, leading to an improved conversion. In addition, the citric acid conversion of 97.7% could still be achieved by PV-esterification at a low reaction temperature of 388 K.

## 1. Introduction

Tributyl citrate (TCB) is considered as a new environment-friendly plasticizer due to its non-toxic, tasteless, high thermal stability and good plasticizing effect, which is widely used in cosmetics, food and medical industries [1,2,3]. TCB was mainly produced by the esterification of citric acid and n-butanol, with concentrated sulfuric acid or solid acid as catalysts [4]. As this reaction was a reversible reaction controlled by thermodynamics [5,6], the maximum yield of TCB was often obtained by using excess n-butanol or the timely removal of by-product water. Compared with the cost increase and subsequent separation difficulty caused by adding excessive n-butanol [7], the timely removal of by-product water could not only improve the reaction conversion but also avoid the inactivation of solid acid catalyst. However, the traditional water removal method by distillation had disadvantages of high energy consumption or complex equipment [8].

Pervaporation (PV) separation technology has the advantages of high selectivity, energy saving and environmental protection, which is widely used in organic solvent separation [9,10,11,12,13], gas separation [14,15] and seawater desalination [16]. The coupling of the membrane reactor with esterification by PV separation technology shows an attractive application prospect [17,18]. At present, some literatures reported that zeolite membrane reactors, such as NaA, CHA, MOR and ZSM-5, were applied to esterification, remarkedly improving the esterification conversion [19,20,21,22,23,24,25]. Li et al. [22] coupled NaA zeolite membrane with the esterification of acetic acid and n-propanol for dehydration through VP separation technology. At 373 K, the final conversion increased from 78.2% to 98.6% within 420 min, when the molar ratio of alcohol/acid was 2:1. Hasegawa et al. [20] applied CHA zeolite membrane to the esterification of adipic acid and isopropanol with concentrated sulfuric acid as catalyst. The membrane was placed in the gas phase to avoid direct contact with sulfuric acid, and the yield of diisopropyl adipate increased from 56% to 98%.

Generally, NaA and chabazite zeolite membranes with a low Si/Al ratio show low acid resistance. The membranes are always placed in the gas phase because they cannot come into contact with acid material directly in the esterification reaction of acid system. This reaction often requires a higher temperature, resulting in unnecessary energy consumption and higher equipment requirements. MOR and ZSM-5 zeolite membranes with a high Si/Al ratio exhibit outstanding acid resistance, but their permeation fluxes are relatively low. Beta zeolite membrane with a three-dimensional twelve-ring cross-pore structure possesses good thermal stability, acid stability and chemical stability, which has a broad application prospect in the fields of organic separation and membrane catalysis [26,27]. The Al-rich beta zeolite membrane with a moderate Si/Al ratio exhibits excellent hydrophilicity and a certain acid resistance, showing great potential in the dehydration of n-butanol mixtures. Therefore, the coupling application of Al-rich beta zeolite membrane and the esterification of citric acid and n-butanol has potential prospects.

In this work, a dense beta zeolite membrane with high selectivity was applied to the esterification of citric acid and n-butanol for the first time. The influence of different catalysts on esterification, with or without PV, was investigated in detail. As a comparison, the coupling of different types of zeolite membrane materials on the esterification was investigated. The effect of different reaction temperatures on the esterification was also studied. In addition, the changes of zeolite membranes before and after esterification were further analyzed by XRD and SEM characterization.

## 2. Experimental

### 2.1. Materials

Beta, NaA, MOR and chabazite zeolite membranes were home-made in our laboratory. All these membranes were synthesized on porous tubular supports by secondary growth method, the molar ratios of synthesis gel and synthesis processes were described in our previous studies [28,29,30,31]. Beta zeolite membrane was prepared in a synthesis gel with a molar ratio of 1SiO_2_: 0.025Al_2_O_3_: 0.3Na_2_O: 0.6NaF: 20H_2_O. N-butanol (purity ≥ 99.5 wt%), potassium hydroxide (purity ≥ 85 wt%) and ethanol (purity ≥ 99.7 wt%) were supplied by Xilong Science Co., Ltd. (Shantou, China). Citric acid monohydrate (purity ≥ 99.5 wt%), p-Toluenesulfonic acid monohydrate (PTSA) (purity ≥ 98.5 wt%), phenolphthalein (pH indicator), and petroleum ether (AR, bp 333–363 K) were provided by Aladdin Company (Shanghai, China). Concentrated H_2_SO_4_ (18.4 mol/L) was produced by Nanchang Xinguang Fine Chemical Factory (Nanchang, China). Anhydrous sodium bisulfate (NaHSO_4_) (purity 95 wt%) was supplied by McLean Inc. (Shanghai, China).

### 2.2. Esterification with PV

The PV-esterification apparatus was depicted in detail in our previous work [25]. Citric acid monohydrate and n-butanol were added to the 150 mL three-neck flask equipped with an agitator and a condensing tube, and was heated to 323 K. After the citric acid monohydrate was completely dissolved, the initial acid value *X*_0_ of the esterification was measured by acid-alkali neutralization titration. Then, heating until the reaction temperature was reached, the timing began when the catalyst and tubular beta zeolite membrane were added. One end of the tubular membrane was plugged and the other end was connected to the vacuum pipeline through a glass tube. The volatilization of reactants was prevented by a reflux condenser installed at the edge of the three-necked flask. Two cold traps were used to collect the permeate by liquid nitrogen. The acid value *X*_n_ was tested every hour. When the change in acid value was small, the reaction was stopped and the final acid value was determined. During the reaction, the whole membrane was completely immersed in the reaction solution and the ratio of effective membrane area to initial reaction solution (S/m) was 0.025 m^2^ kg^−1^. The pressure on the permeate side was controlled below 150 Pa by the vacuum pump. The reaction temperature was kept by using a thermostatic oil bath. The gas chromatography (GC-2014C, Shimadzu, Kyoto, Japan) equipped with a TCD detector was used to analyze the composition of the permeate. The steps of acid-alkali neutralization titration and the calculation of esterification conversion rate were as below.

0.50 g of sample was weighed into a conical flask, and then 25 mL of petroleum ether-ethanol solution was added, with 1–2 drops of phenolphthalein as an indicator. 0.1 mol/L potassium hydroxide-ethanol standard solution was used for titration neutralization to a slight pink color. The titration process should be completed within 30 s until the slight pink color appeared and remained unchanged for 5 s. The acid value was determined by recording the volume change of the standard solution of potassium hydroxide-ethanol. The calculation of acid value was carried out according to the China Standard GB/T1668-2008, which was defined by equations as follows:

$$X=\frac{\left(V-V_{0}\right)\times c\times M}{m}$$

where *X* was the acid value of sample, mg KOH/g; *V* was the volume of potassium hydroxide-ethanol standard solution consumed by the sample, ml; *V*_0_ was the volume of potassium hydroxide-ethanol standard solution consumed by the blank sample, ml; *c* was the concentration of potassium hydroxide-ethanol standard solution, mol/L; *M* was the molar mass of potassium hydroxide [*M* = 56.11], g/mol; and *m* was the weight of sample, g.

The conversion rate of esterification was calculated according to the change of acid value as follows in equation [32]:

$$\eta_{n}=\left(1-\frac{X_{n}}{X_{0}}\right)\times 100\%$$

where *η*_n_ was the conversion rate of sample; *X*_n_ was the acid value of sample, mg KOH/g; and *X*_0_ was the initial acid value, mg KOH/g.

### 2.3. Characterization and PV Test

The crystal phase and crystallinity of zeolite membranes were measured by X-ray diffraction (XRD, Ultima IV, Rigaku, Japan) with a Cu-K*α* radiation in the 2*θ* range of 5–45°. The surface morphologies of the membranes were characterized by a cold field emission scanning electron microscopy (FE-SEM, SU8020, Hitachi, Japan) with an acceleration voltage of 5 kV.

PV performances of zeolite membranes toward a 90 wt% n-BuOH/H_2_O mixture at 348 K were tested by a previously reported PV experimental device [28,29,30,31,33]. The compositions of the feed and permeate were analyzed using a gas chromatograph. The permeation flux (*J*) was calculated according to *J* = m/(A × t), where m, A and t were the weight of permeate (kg), the effective area of membrane (m^2^) and the test time (h), respectively. The separation factor (α) was defined according to α = (Y_i_/Y_j_)/(X_i_/X_j_), where X_i_, X_j_, Y_i_, and Y_j_ were the weight fractions of the components i (water) and j (n-butanol) in the feed and permeate, respectively.

## 3. Results and Discussion

### 3.1. Effect of Catalyst on Esterification without PV

**Figure 1:**
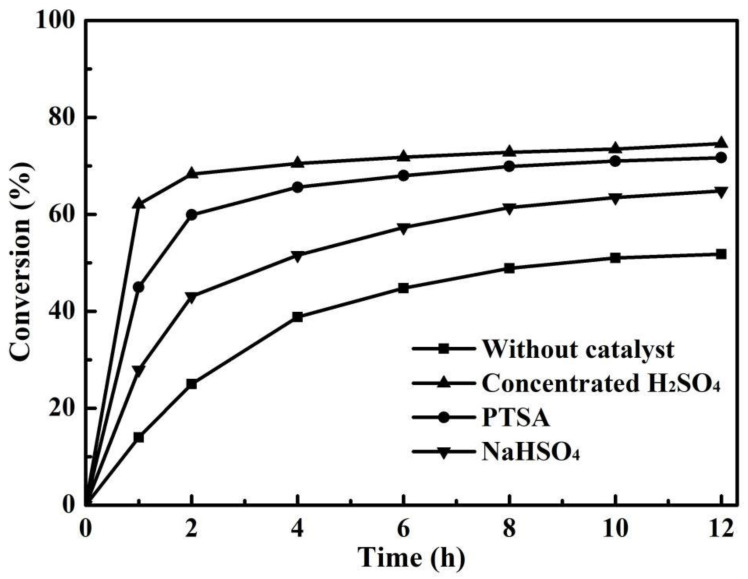
Effect of catalysts on the conversion of citric acid for the esterification without PV (reaction temperature 403 K, catalyst loading 0.7 wt% and initial acid/alcohol molar ratio 1:5).

Esterification reaction is reversible and generally very slow. Without the adding of a catalyst, the conversion is low even after a long reaction time. Thus, the catalyst is very critical to esterification reaction [34]. In this work, three kinds of catalysts including concentrated H_2_SO_4_, p-Toluenesulfonic acid (PTSA) and NaHSO_4_, were used to improve the esterification of citric acid and n-butanol. Figure 1 shows the effects of different catalysts on the conversion of citric acid for the esterification without PV. For comparison, the esterification of citric acid and n-butanol without the catalyst was investigated under the same conditions, as shown in Figure 1. As expected, the reaction without a catalyst was slow, and the conversion of citric acid was only 14.0% after 1 h of reaction. With the extension of reaction time, the conversion of citric acid increased slowly and reached 38.8% after 4 h of reaction. Even after 12 h of reaction, the low citric acid conversion of 51.8% was obtained. By adding a catalyst, the reaction rate and final conversion significantly enhanced. When using NaHSO_4_ as the catalyst, the conversion of citric acid increased to 27.9% after 1 h of reaction. The conversion of citric acid improved to 45% with PTSA as the catalyst, and 62.1% with concentrated H_2_SO_4_ as the catalyst. With the progress of the reaction, the esterification reaction tended to be balanced at 12 h, and the final conversions of citric acid with the three catalysts were 64.8%, 71.7% and 74.6%, respectively. Obviously, as a homogeneous acid, the catalytic activity of concentrated H_2_SO_4_ was higher than that of the heterogeneous acids due to the fact that it could provide much more acidic (hydrogen ions) [25].

### 3.2. Effect of Catalyst on Esterification with PV

**Figure 2:**
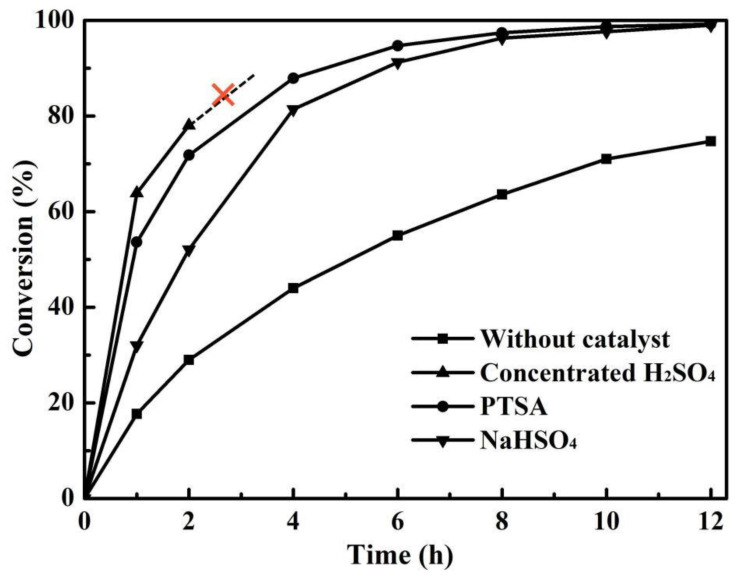
Effect of catalysts on the conversion of citric acid for the esterification with PV (reaction temperature 403 K, catalyst loading 0.7 wt% and initial acid/alcohol molar ratio 1:5).

**Figure 3:**
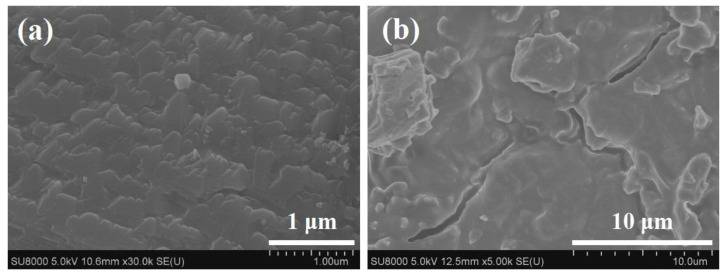
Surface SEM images of beta zeolite membrane before and after PV-esterification using concentrated H_2_SO_4_ as catalyst: (**a**) before and (**b**) after.

**Figure 4:**
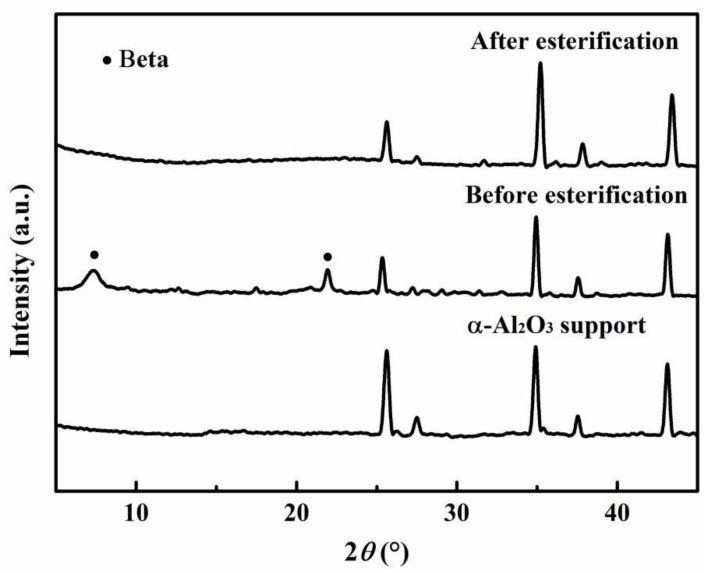
XRD pattern of beta zeolite membrane before and after PV-esterification using concentrated H_2_SO_4_ as catalyst.

The Al-rich beta zeolite membrane was firstly coupled with esterification for continuous dehydration in the esterification of citric acid and n-butanol. The influence of different catalysts on esterification with PV was studied using a beta zeolite membrane under the same reaction conditions of Figure 1. The variation of citric acid conversion with different catalysts versus time is presented in Figure 2. In the absence of a catalyst, once the beta zeolite membrane was introduced for PV-esterification, the final conversion of citric acid was 74.7%, which was greatly higher than that of 51.8% without zeolite membrane, indicating that the removal of water had a significant effect on the esterification reaction. Similarly, when the reaction lasted for 1 h, the citric acid conversion with concentrated H_2_SO_4_, PTSA and NaHSO_4_ catalysts increased to 63.9%, 53.6% and 32%, respectively, which was consistent with the catalytic activity of the catalysts. Unfortunately, when concentrated H_2_SO_4_ was used as the catalyst for PV-esterification, the beta zeolite membrane leaked after 4 h of reaction. The substances on the permeate side of the membrane have been stratified, and gas phase analysis showed that most of the upper liquid was n-butanol. It was speculated that the beta zeolite membrane was dissolved by strong acidic concentrated H_2_SO_4_. The membrane was damaged at 4 h and lost its separation performance. The membrane after PV-esterification with a concentrated H_2_SO_4_ catalyst was further characterized by XRD and SEM, and compared with the membrane before esterification. As observed in Figure 3a, the membrane surface before PV-esterification was covered with a well-crystallized and dense beta zeolite membrane layer. However, these well inter-grown massive polycrystalline particles disappeared on the membrane surface after PV-esterification, and there were some large defects such as cracks on the membrane surface as shown in Figure 3b. By comparing the XRD patterns before and after PV-esterification, it was found that the characteristic peak intensity of the membrane after PV-esterification apparently disappeared (as shown in Figure 4). These results suggested that the beta zeolite membrane could not withstand the strong acidity of concentrated H_2_SO_4_, and the crystal on the membrane surface was destroyed at 4 h.

Nevertheless, beta zeolite membranes showed good acid resistance and stability on PV-esterification with the PTSA and NaHSO_4_ catalysts. As shown in Figure 2, the citric acid conversion with the PTSA catalyst was 97.4% at 8 h and a high conversion of 99.2% was obtained at 12 h. The final conversion of citric acid using the NaHSO_4_ catalyst also achieved 99%. Moreover, at the early stage of reaction, the citric acid conversion with the NaHSO_4_ catalyst was lower than that with the PTSA catalyst, which might be related to the slow dissolution of granular NaHSO_4_. Compared with the powdered PTSA, the NaHSO_4_ was not completely dissolved in the reaction mixture at the initial stage of the reaction, resulting in a low catalytic effect. More importantly, although PTSA was slightly less acidic than the concentrated H_2_SO_4_, it was still a strong acid and its catalytic activity was much higher than that of NaHSO_4_. At the later stage of the reaction, the continuous removal of by-product water in the reaction mixture, with the help of PV technology, also greatly improved the citric acid conversion with the NaHSO_4_ catalyst. In this work, PTSA was considered as the best catalyst for PV-esterification of citric acid and n-butanol.

**Figure 5:**
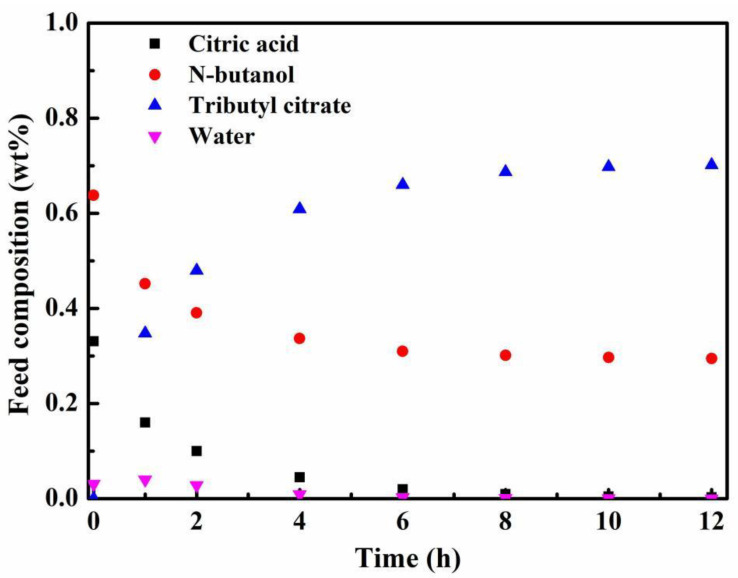
Variation of feed composition with time on the PV-esterification of citric acid and n-butanol (reaction temperature 403 K, PTSA catalyst loading 0.7 wt% and initial acid/alcohol molar ratio 1:5).

Figure 5 illustrates the relationship between the mass fraction of feed component and time on the PV-esterification. As shown in Figure 5, the contents of citric acid and n-butanol decreased rapidly with reaction time until the reactants were almost completely consumed. Due to the excessive use of n-butanol to improve conversion in this work, a certain amount of n-butanol remained in the feed component. The content of tributyl citrate increased rapidly at first and then increased slowly. The initial reactants contained a small amount of water, which was attributed to the fact that the citric acid and the PTSA were both monohydrates, and their crystalline water would be dissolved in the reaction mixture when the substances were dissolved. During the whole reaction process, the water content in the reactant always remained quite low due to the continuous removal of water through the membrane. Finally, a total of 9.3 g of water was removed during the entire PV process, which was close to the theoretical value of 9.41 g.

### 3.3. Effect of Zeolite Membrane Type

**Figure 6:**
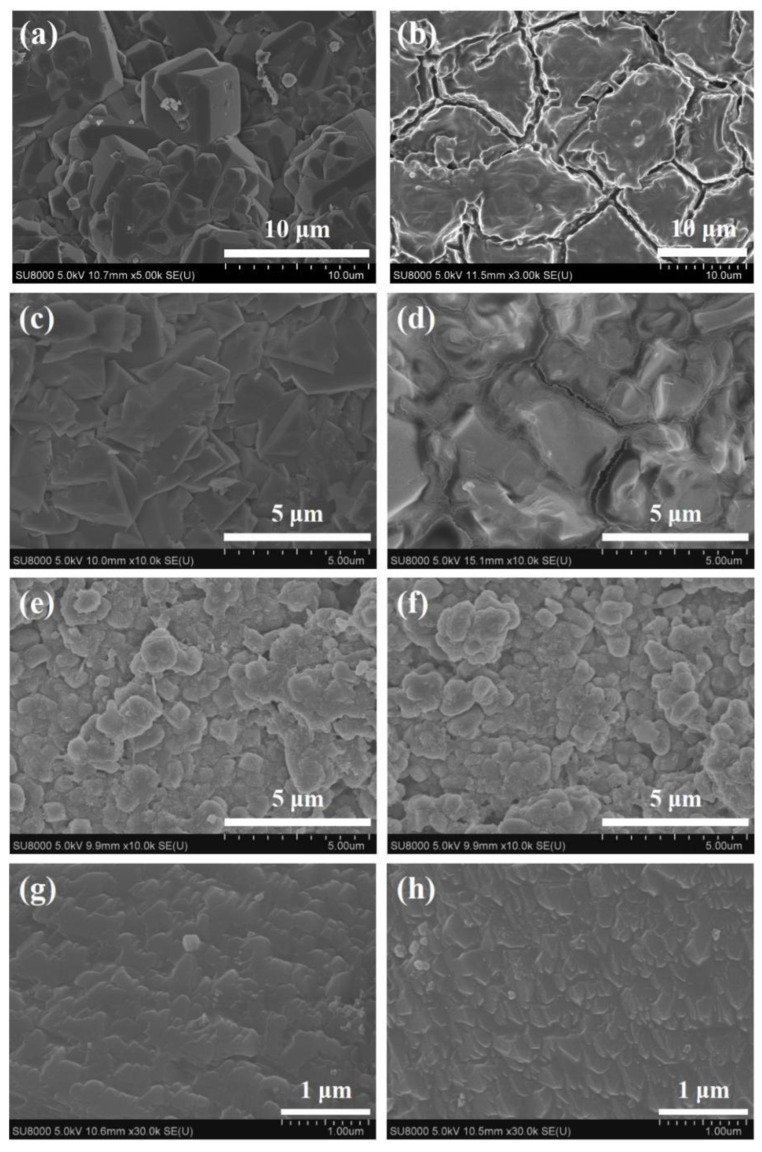
Surface SEM images of different zeolite membranes before and after PV-esterification: NaA zeolite membrane (**a**) before and (**b**) after, chabazite zeolite membrane (**c**) before and (**d**) after, MOR zeolite membrane (**e**) before and (**f**) after, beta zeolite membrane (**g**) before and (**h**) after.

In the PV-esterification process, high water-flux and acid resistance of zeolite membrane are key factors to improve the conversion. In this work, different types of zeolite membranes including NaA, chabazite, beta and MOR were applied to couple with the esterification of citric acid and n-butanol using PTSA as catalyst. Under the same esterification conditions, NaA zeolite membrane lost the separation selectivity after 10 min of reaction, and a large amount of reaction mixture appeared on the permeate side. A similar phenomenon could be detected with using chabazite zeolite membrane after 1 h of reaction. It could be observed from the surface SEM images in Figure 6b,d that the crystals were dissolved and a large number of defects, such as cracks, appeared on the surfaces of NaA and chabazite zeolite membranes after PV-esterification, which made the membranes lose their separation performances. These results indicated that NaA and chabazite zeolite membranes with a low Si/Al ratio were easily dissolved by acid and showed poor acid resistance. The acid resistance of the chabazite zeolite membrane was slightly stronger than that of the NaA zeolite membrane. In the whole PV-esterification process, there was no leakage of beta and MOR zeolite membranes. As shown in Figure 6e–h, the surface of the two membranes basically did not change before and after esterification, and the membrane layers were still dense. This suggested that beta and MOR zeolite membranes had good acid resistance under the acidic conditions of citric acid and PTSA, which could achieve continuous removal of the by-product water from the reaction.

**Figure 7:**
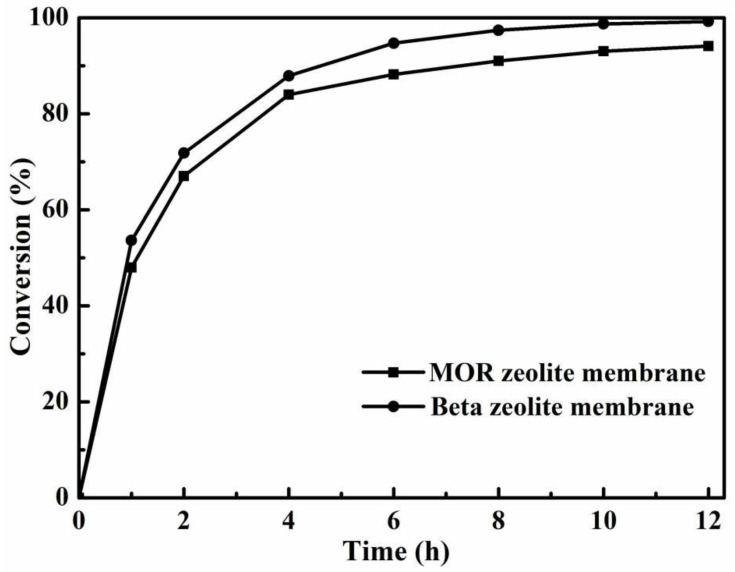
Effect of zeolite membranes on the conversion of citric acid for the esterification with PV (reaction temperature 403 K, PTSA catalyst loading 0.7 wt% and initial acid/alcohol molar ratio 1:5).

Figure 7 presents the conversions of citric acid for the PV-esterification using beta and MOR zeolite membranes. With the use of MOR zeolite membrane, the final conversion of citric acid was 94.1%, which went up by 22.4% compared to the esterification in the absence of zeolite membrane. Meanwhile, the conversion of citric acid obtained by PV-esterification using beta zeolite membrane was always higher than that using MOR zeolite membrane. The high conversion of citric acid using a beta zeolite membrane might be related to the high water-flux of the membrane in the reaction solution. Table 1 shows the dehydration performances of beta and MOR zeolite membranes for a 90 wt% n-BuOH/H_2_O mixture at 348 K. Two kinds of membranes both showed good separation selectivity for n-butanol and water. Water content in permeate of beta and MOR zeolite membranes were 99.94 and 99.83 wt%, respectively. However, the permeation flux of MOR zeolite membrane was only 1.85 kg m^−2^ h^−1^. The beta zeolite membrane with a permeation flux of 2.83 kg m^−2^ h^−1^ was much higher than that of MOR zeolite membrane. It was precisely because of the high permeation flux and excellent separation selectivity of beta zeolite membrane that it had a stronger ability to remove water on the PV-esterification. This was consistent with the trend in Figure 7 that the conversion of citric acid on the beta zeolite membrane was always higher than on the MOR zeolite membrane. This indicated that higher dehydration property of beta zeolite membrane had a stronger promoting effect on esterification, leading to a higher conversion.

### 3.4. Effect of Temperature

**Figure 8:**
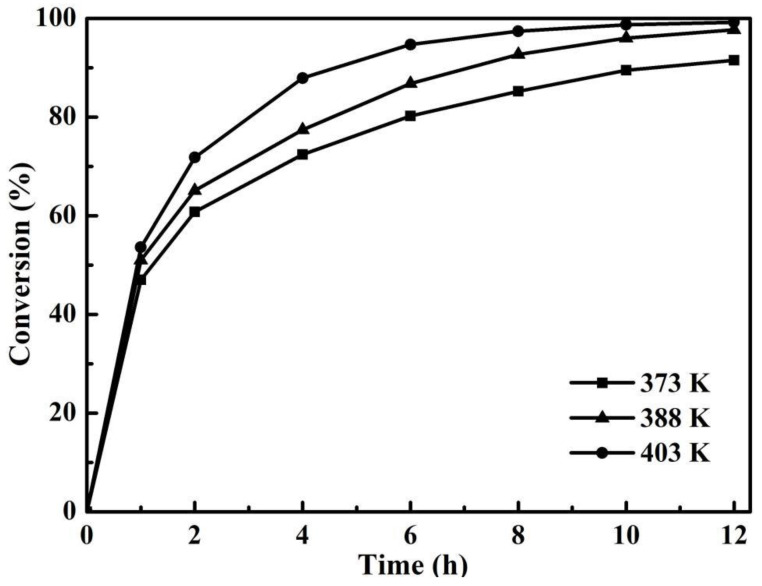
Effect of temperatures on the conversion of citric acid for the esterification with PV (PTSA catalyst loading 0.7 wt% and initial acid/alcohol molar ratio 1:5).

Temperature is an important factor affecting esterification reaction [35]. Since esterification is a reversible reaction, in order to increase the yield of esterification, the reaction must be as much as possible in the direction of ester generation, thus the temperature of esterification is often increased. Generally, the traditional esterification of citric acid with n-butanol usually required a high temperature of more than 403 K to obtain a high yield [36]. In this work, high conversion could be achieved by PV-assisted esterification at a low reaction temperature due to the continuous removal of water through the beta zeolite membrane. Therefore, the esterification of citric acid and n-butanol at the low temperatures of 373, 388 and 403 K are discussed, as shown in Figure 8. It could be seen from Figure 8 that the conversion of citric acid gradually enhanced with the increase of reaction temperature due to the gradual acceleration of reaction speed. After 1 h of reaction, the conversion of citric acid reached 47.0% at 373 K, and improved to 51% and 53.6% at 338 and 403 K, respectively. With the extension of reaction time, the reaction rates and the conversion of citric acid at 403 K were always higher than those at other two temperatures. The conversion of citric acid at 403 K reached 97.4% at 8 h, while the conversion of citric acid at 373 and 388 K were 85.2% and 92.7%, respectively. After 12 h of reaction, the final conversion of citric acid was as high as 99.2% at 403 K, which was higher than that obtained by the water separator method reported in the literature [37]. The final esterification of citric acid at 388 K also reached 97.7%. Even at 373 K, the final esterification of citric acid was 91.5%. Significantly, at the low temperature of 373 K, the water separator method was not effective for the dehydration of esterification process, while PV-assisted esterification could still be adopted to improve the esterification rate.

**Figure 9:**
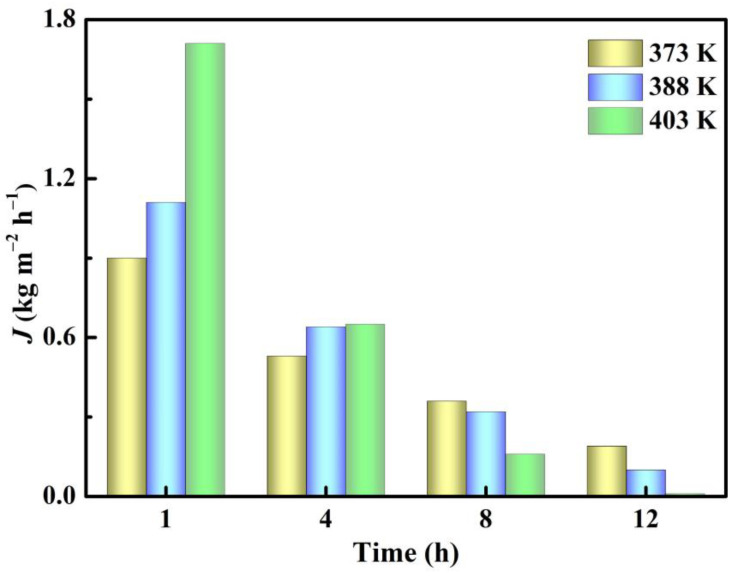
Dependence on time of water permeation flux for the esterification with PV of citric acid and n-butanol under different reaction temperatures.

Figure 9 shows the effect of temperatures at 1, 4, 8 and 12 h on water permeation flux for the esterification with PV of citric acid and n-butanol. Consistent with the results in Figure 8, the increase of reaction temperature accelerated the reaction rate, leading to the formation of more by-product water. In addition, the dehydration performance of beta zeolite membrane in PV process improved greatly with the increase of reaction temperature due to the increasing diffusion rate of H_2_O molecule. As shown in Figure 9, the water permeation flux at 1 and 4 h increased with the increase of temperature. Especially at 1 h, the permeation flux of water at 403 K was much higher than that at the other two temperatures. Water permeation was hardly observed at 403 K at 12 h, because the reaction at a high temperature was fast and water was produced and removed earlier. This was confirmed by Figure 8, showing that the conversion of citric acid at 403 K reached 97.4% at only 8 h, while the conversion at 388 K reached 97.7% and needed 12 h.

## 4. Conclusions

A high-flux and excellent-selectivity beta zeolite membrane was firstly coupled with esterification for continuous dehydration in the esterification of citric acid and n-butanol. Three kinds of catalysts including concentrated H_2_SO_4_, PTSA and NaHSO_4_ were applied in the esterification with or without PV. Compared with the esterification without beta zeolite membrane, the PV-esterification using the PTSA and NaHSO_4_ catalysts, with the help of PV, was greatly improved. When PV-esterification used the concentrated H_2_SO_4_ catalyst, the destruction of membrane surface crystals at 4 h was confirmed by XRD and SEM, indicating that beta zeolite membrane could not withstand the strong acidity of concentrated H_2_SO_4_. With using PTSA as catalyst instead of concentrated H_2_SO_4_, a high citric acid conversion of 99.2% was obtained for the PV-esterification under the same conditions. The comparison of the coupling of different zeolite membrane types on esterification showed that beta zeolite membranes had good acid resistance and high permeation flux. The citric acid conversion of 91.5% was obtained even at a low temperature of 373 K, owing to the continuous water removal on beta zeolite membrane. The conversion of citric acid enhanced from 91.5% to 99.2% when the reaction temperature increased from 373 to 403 K at 12 h.

## Figures and Tables

**Table 1:** PV performances of beta and MOR zeolite membranes for dehydration of a 90 wt% n-BuOH/H_2_O mixture at 348 K.

Zeolite Membrane	*J* (kg m^−2^ h^−1^)	*α*H_2_O_/_n-BuOH	Water Content in Permeate (wt%)
beta	2.83	15,000	99.94
MOR	1.85	5290	99.83

## Data Availability

Not Applicable.
